# 25-Hydroxyvitamin D Status and Its Predictors in Greek and Cypriot Subsets of the UK Biobank Cohort

**DOI:** 10.3390/nu17203267

**Published:** 2025-10-17

**Authors:** Francesca E. Kontea, Susan A. Lanham-New, Andrea L. Darling

**Affiliations:** 1 Discipline of Nutrition, Exercise, Chronobiology and Sleep, School of Biosciences, Faculty of Health and Medical Sciences, University of Surrey, Guildford GU2 7XH, UK; 2 Anderson’s by Francesca Kontea (Private Nutrition & Dietetics Consultancy), Doriza 10A, 11525 Athens, Greece

**Keywords:** 25-hydroxyvitamin D, ethnicity, epidemiology, cohort

## Abstract

**Objective:** Studies show a high prevalence of vitamin D deficiency in Greece and Cyprus despite an abundance of sunlight. We investigate the vitamin D status of Greeks and Cypriots living in the UK, where sunlight availability is more limited. **Design:** Cross-sectional study of serum 25-hydroxyvitamin D (25(OH)D) using the UK Biobank cohort. **Setting:** The UK Biobank is a study of over 500K UK dwelling participants, with baseline measurements from 2006–2010. **Participants:** A sample of 325 Greek/Cypriot and 4158 British/Irish participants (aged 40–69 years). **Results:** The Greeks/Cypriots had statistically significantly lower median serum 25-hydroxyvitamin D (25(OH)D) (40.3 nmol/L) compared to the British/Irish (47.6 nmol/L). Eleven percent of British/Irish and 22.8% of Greeks/Cypriots had serum 25(OH)D < 25 nmol/L. Being exposed to summer sunlight for >30 min/d, as well as having a blood draw in summer or autumn, was statistically significantly associated with lower odds of 25 (OH))D < 50 nmol/L. Living in Scotland, having a winter blood draw, and not using a vitamin D-containing supplement were associated with increased odds of 25(OH)D < 50 nmol/L. Ethnicity was not a predictor of 25(OH)D < 50 nmol/L after confounder adjustment (Greek/Cypriot OR = 1.18 (95% CI 0.85, 1.63; British/Irish OR = 1.0). **Conclusions:** UK dwelling Greeks/Cypriots have a higher prevalence of vitamin D deficiency (<25 nmol/L) compared to the British/Irish population, but evidence from the literature is mixed as to whether they have a higher prevalence than when living in their country of origin. Public health interventions are required to improve 25(OH)D status in UK ethnic minority groups.

## 1. Introduction

Vitamin D deficiency has been shown to play a key role in many health problems such as osteoporosis, cancer and autoimmune diseases, diabetes, metabolic syndrome and blood pressure, all of which currently pose worldwide public health challenges [1].

Geographical location is a key factor influencing production of vitamin D in the skin [2]. Latitudes between 37° N–37° S receive enough UVB exposure for skin to produce vitamin D year-round. However, for latitudes above 37° N and below 37° S, vitamin D synthesis is compromised during the winter months due to insufficient UVB radiation, and there is reliance on nutritional intake of vitamin D [2,3].

Multiple studies have shown that vitamin D deficiency is a widespread public health issue throughout Europe [4,5,6,7,8,9]. Vitamin D deficiency was originally considered a problem of northern European countries, whereas the sunnier countries of southern Europe were thought to be vitamin D sufficient due to higher UVB availability [10]. However, data now show that vitamin D deficiency is actually more prevalent in southern European countries [11]. For example, a study of 7441 postmenopausal women from 29 countries around the world found that Northern European countries had a higher 25(OH)D level than did Southern European countries [12]. Some studies have investigated 25(OH)D levels in individuals from European countries and found that southern European countries have the lowest concentrations, for example, Greece (between 25 and 31 nmol/L), Portugal (39 nmol/L) and Italy (32 nmol/L) [10,13,14]. In one review of global 25(OH)D concentrations, the lowest European 25(OH)D concentrations were found in Greece (30 nmol/L), the UK (29 nmol/L) and Italy (45 nmol/L), whereas the highest were found in countries such as Norway (75 nmol/L) and Sweden (69 nmol) [13]. This difference has been attributed to the fact that far northern European countries traditionally have a higher consumption of oily fish and cod liver oil, and the lower 25(OH)D concentrations found in Spain, Italy and Greece may be due possibly to darker skin pigmentation and sun-avoidant lifestyle [13,15]. Some far northern European countries also have robust vitamin D fortification policies [16].

In Greece specifically, an increasing number of studies are now confirming that vitamin D deficiency is a widespread problem, with studies finding a high prevalence of 25(OH)D deficiency in exclusively breast-fed neonates [17] as well as pregnant women [18], children and adolescents [19]. Of high concern, there are still cases in Greece of rickets in both infants and young children [20,21].

There are more limited data on the vitamin D status of Cypriots. Some studies, conducted in Cyprus, have shown widespread vitamin D deficiency in adolescents, with up to one in three children deficient and predictors of deficiency including having darker skin, less skin exposure during winter, having a higher BMI and being of female gender [22,23]. A study investigating vitamin D status in 8780 Greeks from Thessaloniki, Greece (40.7° N) and 2594 Cypriots from Nicosia, Cyprus (35.1° N) found that 73.1% of Greeks and 69.8% of Cypriots had low 25(OH)D concentrations at 62.7 nmol/L and 63.4 nmol/L, respectively [24].

In terms of immigration, numerous studies illustrate that populations moving from nearer the equator to more northerly latitudes are at increased risk of vitamin D deficiency [25,26,27,28,29]. For example, studies on individuals originating from South Asia, Turkey and Morocco, now living in countries such as Norway and the UK, show that these ethnic groups are at very high risk of vitamin D deficiency [25,26,27,28,29]. With a high prevalence of vitamin D deficiency in Greece and Cyprus, where sunlight is plentiful, it can be speculated that 25(OH)D concentrations in these populations would be even lower when moving to a higher-latitude country, with lower UVB radiation during the winter and known high prevalence of deficiency amongst ethnic minority groups. It is therefore important to investigate vitamin D status in Greeks/Cypriots living in higher-latitude countries, as well as assess variables that may be associated with 25(OH)D concentration for this population group.

Therefore, we assessed whether there are any differences between Greeks/Cypriots (GC) born in Greece or Cyprus (and living in the UK) and their British/Irish (BI) peers, regarding 25(OH)D status; dietary and supplemental vitamin D intake; anthropometric measurements; and demographic and social variables. We also assessed how these individual variables relate to each ethnic group’s serum 25(OH)D concentrations.

## 2. Methods

### 2.1. UK Biobank Cohort

The UK Biobank is a population-based prospective cohort study involving over 500,000 participants aged 40–69 years at baseline, recruited though NHS primary care patient lists between 2006 and 2010 [30]. It includes data from dietary and lifestyle questionnaires and physical and biochemical measurements, as well as genetic data [30]. Participants were recruited from across England, Wales and Scotland, excepting the North of Scotland, the Southwest of England and the East of England [30]. Latitudes included ranged from Reading in the South of England (51° N) to Edinburgh in Scotland (56° N) [30].

We present a cross-sectional analysis of a 4483 UK Biobank participants aged 40–69 years at baseline. Our analysis includes 140 Greeks and 185 Cypriots, of self-reported ethnicity, born in Greece or Cyprus but living in the UK. As a comparison group, we included a sample of 4158 English and Irish participants, also from the UK Biobank, randomly selected from the White subsample of the cohort using IBM SPSS Statistics for Windows, version 28 (IBM Corp., Armonk, NY, USA). No other inclusion criteria were used other than ethnicity.

We were unable to discern Cypriot ancestry using solely self-reported ethnicity, as many of those of Cypriot ancestry (born in Cyprus but living in the UK), listed themselves as British or European. Therefore, using data for genetic principal components, participants born in Cyprus but highly likely to be of British heritage were removed from the analysis, as they likely represented family of British Military personnel rather than individuals of Cypriot ancestry.

### 2.2. Derivation of Variables

All variables reported here were from baseline measurements only, unless otherwise stated. For assessment of serum 25(OH)D concentration, non-fasted blood samples were drawn once from each participant by UK Biobank staff. Therefore, each participant had a 25(OH)D measurement in one season only, and this could have been drawn in any one of the four seasons. Serum 25(OH)D measurements were conducted using the DiaSorin Liaison XL assay, (DiaSorin S.p.A, Saluggia, Italy) which measures 25(OH)D_2_ and 25(OH)D_3_ and is a direct competitive chemiluminescent immunoassay [31]. Of note, when using this assay, 25-hydroxyvitamin D levels at 25 nmol/L may be underestimated by 4%, and serum levels above 40 nmol/L may be overestimated by 5–10%, whilst the lowest point of detection is 10 nmol/L [32]. A similar percentage (9%) had missing data for both BI and GC. Value being outside assay limits applied to 0.5% of British/Irish and 1.5% of Greeks/Cypriots. It was assumed these were highly likely to all be under the lower limit (rather than over the upper limit), so a sub-analysis was undertaken with these missing data points corrected using the standard equation (assay lower limit (10 nmol/L) divided by the square root of 2, giving 7.1 nmol/L). However, this had no meaningful impact on the data, so the data are not shown.

The Oxford WebQ 24 h recall food frequency questionnaire (FFQ) was used to assess vitamin D intake. This questionnaire assesses how often certain food items were consumed in the last 24 h [33]. This was completed once during the baseline visit via touchscreen and subsequently, participants that had provided an email address were invited to complete the questionnaire up to another 4 times online during the time period of February 2011–April 2012 [33]. Forty-one per cent of Greeks/Cypriots and 43% of British/Irish had at least one completed dietary recall. Many different food groups were included that covered the main sources of both vitamin D2 and vitamin D3; these were both included together in one result representing total vitamin D intake [33]. Other studies have noted that when compared to other 24 h recall questionnaires, this FFQ compares well in its accuracy in estimating nutrient intake [34]. We calculated median vitamin D intakes from across all completions of the FFQ in order to increase validity of the vitamin D intake. This did not include supplement use, as we did not have data for the dose of the supplements, so we could not add this value to that of the vitamin D intake from food. Vitamin D-containing supplement use was assessed as a separate binary variable (yes/no), as this is the only information available.

Oily fish was assessed during the touchscreen questionnaire, which contained questions about a variety of demographic and general dietary factors [35]. Participants were asked to rate how often they generally usually ate oily fish (not just in the last 24 h), including the following options: never; <once per week; 2–4 times per day; 5–6 times per week; once or more daily; do not know; prefer not to answer [35]. As part of the same questionnaire, participants also self-reported if they took a multivitamin (which we assumed contained vitamin D) or a single vitamin D supplement and those data were then dummy coded to form a new variable for combined intake from either/both [35]. No brand or dosage information was available for vitamin D, and we could not assess cod liver oil supplementation, as the Biobank question for this related to use of both omega 3 fish oil and cod liver oil in the same question, prohibiting separate assessment.

Age at baseline assessment, age that full-time education was completed, household income, skin colour, smoking status, alcohol intake, biological sex, as well as time exposed to summer sunlight were all self-reported in the touchscreen questionnaire. The Townsend Deprivation Index [36]—a measure of population material deprivation, based on unemployment, non-car ownership, non-home ownership and household overcrowding—was derived (by UK Biobank staff) from participant post code. Baseline Body Mass Index (BMI) was assessed by UK Biobank staff using the Tanita BC418MA (Tanita Corporation, Tokyo, Japan) body composition analyser.

### 2.3. Statistical Analysis

All statistical analysis was carried out using IBM SPSS Statistics for Windows, version 28 (IBM Corp., Armonk, NY, USA). Inspection of histograms was used to check for normality in continuous variables. Continuous variables of interest—such as 25(OH)D serum levels, vitamin D intake, Townsend deprivation index, age at baseline assessment, and age completed full-time education— were not normally distributed, so Mann–Whitney tests were used to assess statistical significance by ethnic group. For categorical or ordinal variables—gender, household income, oily fish intake, alcohol consumption, skin colour, smoking status, supplement use, region of assessment, sunlight exposure and season of blood draw—a chi-square test was used to assess associations with ethnicity.

For the logistic regression models, 25(OH)D levels were recoded into a dummy variable for above and below 25 nmol/L and for above and below 50 nmol/L. A chi-square test investigated descriptive characteristics for these two variables, and 25 nmol/L was found to not have a good balance in participants over the two dummy categories, and therefore a logistic regression was only conducted for the cutoff of 50 nmol/L. The logistic regression models included all categorical variables mentioned above. A sub analysis was undertaken separately for the different ethnic groups (British/Irish (BI) and Greek/Cypriot (GC)), as well as for all participants as one group. A power calculation was conducted. For a 5 nmol/L mean difference in 25(OH)D, assuming a standard deviation of 21 nmol/L (based on data at https://biobank.ndph.ox.ac.uk/showcase/field.cgi?id=30890, accessed on 26 September 2022), alpha = 0.05 and power = 80%, *n* = 278 were needed in each group.

## 3. Results

### 3.1. Descriptive Statistics

As shown in Table 1, the BI group was found to have a statistically significantly higher median 25(OH)D at 47.6 nmol/L compared to the GC group at 40.3 nmol/L (<0.001). The groups had a similar vitamin D intake, with GC at 1.4 μg/day and BI at 1.8 μg/day (*p* = 0.045). The median (Interquartile Range; IQR) age of BI participants was 58 (12) years, and for GC participants was 57 (12) years (*p* = 0.004) (statistically significant but not of biological relevance). BI had a statistically significantly lower Townsend deprivation index at −2.31 compared to GC at −0.66, indicating that GC lived in slightly more deprived areas (*p* < 0.001), although absolute deprivation levels were still low in both groups. There was also a statistically significant difference in the age that both ethnic groups completed full-time education, with BI completing full-time education 1 year earlier, at a median age of 16 years, compared to GC with a median of 17 years (*p* < 0.001). This could indicate differences in enrolment in further education or may reflect differences in school leaving age by country. Finally, there was a statistically significant difference in BMI between the two ethnic groups, (*p* < 0.001); however, it was not a clinically relevant difference, with mean BMI for BI at 27.4 kg/m^2^ and for GC at 28.5 kg/m^2^.

#### 3.1.1. Categorial Descriptives by Ethnicity: Sex, Socioeconomic Demographical Lifestyle and Dietary Factors

As can be seen in Table 2, a Chi-square test showed a higher percentage of female participants in the BI group (55.7%) compared to the GC group (47.1%) (*p* = 0.003). Accordingly, the percentage of male participants was higher in GC (52.9%) than in BI (44.3%). There was a statistically significant association between geographical region and ethnicity (*p* = <0.001). The highest proportion of BI participants resided in Northern England (47.3%) followed by Southern England (16.8%) and the Midlands (16%). On the other hand, the largest proportions of GC participants lived in London (47.4%) and Southern England (17.8%). There was also a statistically significant relationship between ethnicity and household income per year (*p* < 0.001), with more BI participants in the income brackets up to £52K, and a higher number of GC in the income ranges above £52K.

Skin colour showed a statistically significant relationship with ethnicity (*p* < 0.001), with the majority of BI reporting to have fair skin compared to the majority of GC describing their skin colour as light olive.

Compared to BI, a higher percentage of GC were current or previous smokers, with BI having a higher number of participants that had never smoked (*p* = <0.001). Conversely, a higher percentage of the BI group consumed alcohol daily (20.7%) compared to only 11.1% of GC the group, with GC having a higher percentage of people only drinking at special occasions or never (*p* = <0.001).

The GC group consumed oily fish more often than did BI (*p* = 0.002). Specifically, a higher proportion of BI (10.8%) reported to never consume oily fish compared to GC (4.4%), and more GC participants had oily fish 5–6 times per week than did BI. GC participants were slightly more likely to consume a vitamin D containing supplement (28.4%) than were BI participants (23.6%), although this result did not quite reach conventional levels of statistical significance (*p* = 0.05).

Both ethnic groups had many participants who reported having more than 30 min sun exposure each day during the summer months, with 95.9% of BI reporting to do so as well as 91.9% of GC. A smaller proportion of BI had low sun exposure, with a lower proportion (4.1%) spending under 30 min a day in the sun as compared to GC (8.1%) (*p* = <0.001). Season of assessment was related to ethnicity, with the highest proportion of participants in both ethnic groups assessed during spring (*p* = <0.001), but the second largest proportion of BI participants assessed in the summer, and for GC the second largest group in autumn. This should be borne in mind when assessing overall serum 25(OH)D status in BI and GC.

#### 3.1.2. Ethnicity and Serum 25(OH)D Status

A chi-square test showed a statistically significant association between ethnicity and serum 25(OH)D levels for the cutoff of 25 nmol/L, as well as for the cutoff of 50 nmol/L (Table 3). For all participants, 11.9% had 25(OH)D levels below 25 nmol/L and 53.8% had levels below 50 nmol/L. However, when looking at the ethnic groups separately, the GC ethnic group had the highest percentage of people below 25 nmol/L (22.8%) as well as the highest proportion of individuals below 50 nmol/L (62.3%).

**Figure 1:**
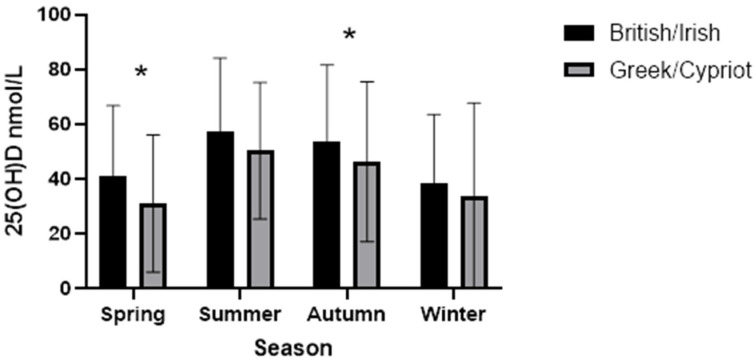
Median (IQR) for 25(OH)D serum levels by season for each ethnic group. Mann–Whitney U Test between ethnic groups, within seasons (* *p* < 0.01 Bonferroni adjusted). Participant numbers: Spring British Irish (BI) = 1040; Summer BI = 983; Autumn BI = 911; Winter BI = 773; Spring Greek/Cypriot (GC) = 102; Summer GC = 59; Autumn GC = 70; Winter GC = 58.

As illustrated in Figure 1, BI achieved much higher median 25(OH)D levels across all seasons than did GC. In both summer and autumn, BI achieved serum levels above 50 nmol/L, but serum levels for GC barely exceeded 50 nmol/L even in the summer. The third-highest levels were seen in spring for BI and winter for GC.

### 3.2. Logistic Regression Model for Prediction of Odds of 25(OH)D Levels > 50 nmol/L

#### 3.2.1. All Participants

Ethnicity was not a significant predictor of 25(OH)D < 50 nmol/L, with the GC group having an OR of 1.18 (95% CI 0.85, 1.63) compared to the BI group (reference category) (*p* = 0.33) (Supplementary Table S1).

Biological sex, household income, oily fish intake, alcohol consumption and smoking status did not show statistical significance. However, supplementation was a statistically significant predictor, with those who did not use a single vitamin D supplement or multivitamin/mineral supplement being 2.4 times more likely (OR. 2.4 (95% CI 2.01, 2.89)) to have a 25(OH)D level of <50 nmol/L compared to those who did use one (*p* < 0.001). Compared with Northern England (reference category), those from Scotland had 1.5 times higher odds of vitamin D deficiency (*p* = 0.009) (OR 1.5 (95% CI 1.1, 2.03)). The South of England, Wales, The Midlands and London were not statistically significantly different compared to those from Northern England. Being exposed to summer sun for >30 min a day was associated with decreased odds of 25(OH)D < 50 nmol/L by 56%, compared to having exposure for less than 30 min a day (*p* < 0.001) (OR 0.44 (95% CI 0.295, 0.65).

Finally, participants who had a blood sample taken during autumn had 68% reduced odds of 25(OH)D < 50 nmol/L, with an OR = 0.32 (95% CI 0.26, 0.40), and those during summer had an even lower odds at 79% (OR = 0.21 (95% CI 0.17, 0.26)) compared to spring (reference category, both *p* < 0.001). Participants with blood drawn during winter showed similar odds of 25(OH)D < 50 nmol/L to that of spring, with an OR = 1.18 (95% CI 0.94, 1.48).

#### 3.2.2. British/Irish Group Only

Sex, household income, oily fish intake, alcohol consumption and smoking status were not statistically significant predictors of odds of having 25(OH)D < 50 nmol/L (Supplementary Table S2). As when run for all participants, the logistic regression for the BI participants showed that a non-user of a vitamin D supplement or multivitamin and mineral supplement had increased odds of 25(OH)D < 50 nmol/L by 2.4 times (OR = 2.40 (95% CI 1.97, 2.78)) (*p* < 0.001).

BI participants living in Scotland (OR 1.44 (95% CI 1.06, 1.95) had 1.44 times higher odds of 25(OH)D levels < 50 nmol/L compared to those from the North of England (reference category, *p* = 0.02). The other areas did not show statistical significance.

Individuals reporting exposure to summer sunlight for >30 min per day were at decreased odds of 25(OH)D < 50 nmol/L by 52% (OR 0.48 (95% CI 0.31, 0.72)) (*p* < 0.001). As with the model for all participants, BI showed lower odds of having 25(OH)D levels < 50 nmol/L when blood draw was in summer and autumn, by 79% and 66%, respectively, compared to the reference category of spring (summer OR 0.21 (95% CI 0.17, 0.26)) (autumn OR 0.33 (95% CI 0.27, 0.41)).

#### 3.2.3. Greek/Cypriot Group Only

As for all participants and for the BI group, the variables of biological sex, household income, oily fish intake and alcohol consumption were not predictors of 25(OH)D < 50 nmol/L for the GC participants (Supplementary Table S3). However, smoking was a statistically significant predictor of 25(OH)D < 50 nmol/L. Specifically, those having previously smoked were 2.4 times more likely to have 25(OH)D < 50 nmol/L compared to those who had never smoked (OR 2.4 (95% CI 1.03, 5.44)).

GC also differed from the previous analyses in that no region of assessment predicted 25(OH)D < 50 nmol/L. On the other hand, in accordance with the previous logistic regression results, non-use of either a single vitamin D supplement or multivitamin/mineral supplement correlated with 3 times the odds of 25(OH)D < 50 nmol/L (OR 3.05 (95% CI 1.47, 3.35)) (*p* = 0.003).

Exposure to summer sun for >30 min per day was associated with decreased odds of 25(OH)D < 50 nmol/L, by 87% (OR 0.125 (95% CI 0.025, 0.63)) (*p* = 0.012). In addition, individuals who had blood samples taken during summer and autumn had 87% and 83% respectively decreased odds of 25(OH)D < 50 nmol/L compared to those with samples taken in spring (summer OR 0.13 (95% CI 0.05, 0.34); autumn OR 0.17 (95% CI 0.07 0.42)).

## 4. Discussion

### 4.1. Summary

We found that serum 25(OH)D concentrations differ by ethnicity, with the British/Irish ethnic group having statistically significantly higher 25(OH)D by 7.3 nmol/L. However, the logistic regression analysis showed that ethnicity was not a significant predictor of vitamin D deficiency (<50 nmol/L), indicating confounding variables related to ethnicity may explain the differences in 25(OH)D between the Greek/Cypriot and British/Irish groups. In terms of predictors of deficiency, being exposed to summer sunlight for more than 30 min a day, as well as blood draw in summer or autumn, was statistically significantly associated with lower odds of 25(OH)D < 50 nmol/L. Additionally, not using a vitamin D-containing supplement, living in Scotland, and winter blood draw were associated with increased odds of vitamin D deficiency. Gender, household income before tax, oily fish intake and alcohol consumption were not predictors of vitamin D deficiency. Smoking status was only a predictor of serum 25(OH)D < 50 nmol/L in the Greek/Cypriot specific analysis.

#### 4.1.1. Serum 25(OH)D Concentration

Our data show that a large proportion of UK dwelling British/Irish and Greek/Cypriot persons do not meet SACN- or EFSA-recommended 25(OH)D levels [37,38], with only 11% of the British/Irish ethnic group and 22.8% of the Greek/Cypriot ethnic group having 25(OH)D > 25 nmol/L, as well as 53.1% of the British/Irish group and 62.8% of the Greek/Cypriot group having 25(OH)D > 50 nmol/L.

To our knowledge, there are no other published data reporting 25(OH)D status for Greeks or Cypriots living in the UK. Therefore, the results of our study can only be compared to data for the rest of the UK population, or to Greeks/Cypriots living in their country of origin. For the latter, some studies have reported 25(OH)D as low as 25–31 nmol/L [10,39], whilst other studies have reported as high as 62.7–63.42 nmol/L [24]. For example, a study conducted in urban adolescents living in Northern Greece found that 47% of those aged 15–18 years and 14% of those aged 13–14 years had wintertime 25(OH)D < 25 nmol/L [40]. In terms of adults, a small study of women (aged 25–50 years, living in Athens) found a high prevalence of vitamin D deficiency, with the women having a low vitamin D intake (<400IU), high sunscreen use all year round and low sunshine exposure outside of the summer months [41]. Similarly, a study of 970 adults (from rural and urban areas) found that up to 87.7% had low vitamin D serum levels, with women having the lowest [42]. In terms of older individuals, one study in Athens (38° N) showed that 20% had severe vitamin D deficiency (<25 nmol/L) and only 6.5% had vitamin D levels above 80 nmol/L [43]. In another study, 58 older participants living in nursing homes showed serum 25(OH)D level of only 19 nmol/L [44].

One study showed 54% of Greek adults had serum levels below 50 nmol/L [42], indicating a lower prevalence of deficiency in the country of origin compared to the UK Greeks/Cypriots in our current study (62.3%). The Food4Me study found that 1.4% of Greeks had 25(OH)D < 30 nmol/L, and 34.8% had 25(OH)D 30–49.9 nmol/L [45]. We found that Greeks/Cypriots had a median 25(OH)D concentration of 40.4 nmol/L which falls within the range of values cited in studies of this group in their country of origin [10,24,39].

#### 4.1.2. Vitamin D Intake

We found that vitamin D intakes were low, at 1.77 μg/day and 1.41 μg/day for British/Irish and Greeks/Cypriots, respectively. These intakes are much lower than the recommendations of SACN (10 μg/day) [38] and EFSA (15 μg/day) [37]. The intakes were also lower (for both British/Irish and Greek/Cypriot groups) than the 2.8 μg/day (for adults aged 19–64 years) and 3.3 μg/day (for adults aged ≥65 years) in the UK National Diet and Nutrition Survey (NDNS) [46]. This discrepancy could be explained by differing methods of dietary assessment. A study in 2218 Greek adults showed that 99% did not meet EFSA’s EAR (10 μg/day) [37], whilst 92–94% did not take a vitamin D-containing supplement [47]. Mean vitamin D intake was 1.0–1.7 mcg/day, the sources of which were 47% from fish; 15% from meat; and 12% from cereal [47]. Similarly, in a study of Greek adults (aged ≥ 19 years) living in Greece, almost 100% did not meet EFSA’s estimated average requirement (EAR) (10 mcg/day) [37], with median intakes of 1.2–1.7 mcg/day and 1.0–1.3 mcg/day, respectively [42]. These are similar to our results for the Greek/Cypriot group.

However, the majority of the UK Biobank participants did not use a vitamin D-containing supplement. Our ethnic specific regression analyses showed that British/Irish participants were 2.4 times more likely, and the Greeks/Cypriots were 3.0 times more likely, to have 25(OH)D < 50 nmol/L if not using a vitamin D-containing supplement. The percentage of Greeks/Cypriots not taking a supplement in our analysis was 71.6%, which was much lower than that found in a study of Greek adults (92–94%) living in Greece. Ref. [47] Data from Greece suggest that supplement use is a predictor of vitamin D sufficiency (≥50 nmol/L), increasing odds by 1.87 times with a supplement of 5.0–9.9 μg/day, 5.5 times with 10–19.9 μg/day and 14.2 times with 20–80 μg/day compared to <2.5 μg/day [45].

We found that the Greek/Cypriot group had a higher oily fish intake than did the British/Irish group, but logistic regression showed that when confounders were controlled for, oily fish intake was not a predictor of deficiency in either ethnic group. These results are unexpected since oily fish is one of the richest natural sources of vitamin D, and its consumption has been routinely shown to be associated with higher vitamin D serum levels in results from other studies [48]. European studies have suggested that the higher 25(OH)D levels found in Northern European countries, such as Norway, could be explained partially due to the higher intake of oily fish compared to that of Southern European countries [39,49]. The majority of participants in both ethnic groups of this current study did not meet recommendations of two portion of oily fish (>140 g/week) [46]. Therefore, the lack of association between vitamin D status and oily fish intake could be explained by the low number of participants consuming adequate amounts of oily fish.

#### 4.1.3. Lifestyle

A higher proportion of British/Irish participants reported to have never smoked compared with the Greek/Cypriot group. The Greek/Cypriot group contained more current or previous smokers, and being a previous smoker was associated with 2.4 times higher odds of having vitamin D levels < 50 nmol/L, compared to never smoking. Other studies have shown a significant association between smoking and vitamin D deficiency in other Southern European populations, with the association restricted to current but not previous smokers [50]. Alcohol consumption was associated with ethnicity, but it was not shown to be a predictor of vitamin D serum levels < 50 nmol/L. A higher proportion of British/Irish participants consumed alcohol daily and more frequently compared to the Greeks/Cypriots.

Exposure to summer sunlight for >30 min/day was a significant predictor in reducing risk of 25(OH)D < 50 nmol/L for all participants. It is known that spending more time outside during the summer months is associated with decreased risk of vitamin D deficiency [2,10,51]. In a study of seven European countries, including the UK and Greece, light to moderate outdoor physical activity for 30–60 min/day was associated with an increased likelihood of sufficiency (>50 nmol/L), by 1.8 times compared to outdoor physical activity of <30 min/day [45].

In our analysis, ethnicity was associated with summer sunlight exposure, with a higher percentage of British/Irish spending > 30 min/day compared to the Greeks/Cypriots. This is not surprising since the Greek/Cypriot participants may have adopted sun-avoiding behaviour due to the high intensity of the summer sun. Sun avoidance in southern European countries has been suggested as a possible reason for decreased vitamin D serum levels in southern countries compared to northern Europe [10,15,52].

#### 4.1.4. Background Demographics

In this study, the majority of British/Irish participants reported fair skin tone, whilst Greeks/Cypriots characterised their skin as light to olive tones. Darker skin pigmentation may help explain lower vitamin D status, due to lower vitamin D production compared to the fairer skin tone more prominent in populations in northern European countries [15]. Therefore, ethnic groups from Southern Europe could be at higher risk of vitamin D deficiency when living in Northern countries [15,53], compared to the fairer skinned population. In this current study, there were more females in the British/Irish group than in the Greek/Cypriot group (55.7% vs. 47.1%). However, sex was not a significant predictor of 25(OH)D < 50 nmol/L. In contrast, several global studies have found that being female is associated with higher risk of being vitamin D deficient [15,54]. Also, data from seven European countries (including UK and Greece), show that women have higher levels of insufficiency (30–49.9 nmol/L) at 5.2% (compared to 0.7% for males) and deficiency (<30 nmol/L) at 36.6% (compared to males at 22.6%) [45].

Studies in Greek populations have shown that women are at higher risk of vitamin D deficiency [24,55]. The UK NDNS (2016–2019) reported that women aged 19–64 years had lower intakes of vitamin D compared to men, even when using a supplement, but had higher intakes of oily fish as well as higher mean levels of 25(OH)D (47.4 nmol/L) [46]. Women aged 65 years and over had lower serum levels compared to same-age men [46].

#### 4.1.5. Geographical and Seasonal Factors

Ethnicity was significantly associated with geographical region, which was shown to be a significant predictor of vitamin D status. For the model including all participants, as well as the British/Irish ethnic group separately, living in Scotland was associated with increased odds of 25(OH)D levels < 50 nmol/L. The same was not found for the Greeks/Cypriots, possibly due to only very small numbers of this group living in Scotland. These results agree with previous literature illustrating lower vitamin D serum levels found in Northern areas of the UK, such as Scotland, compared to Southern regions [46,56].

Season of blood draw differed by ethnicity, as well as being a predictor of vitamin D status. Blood draws in summer and autumn were associated with reduced risk of having 25-hydroxyvitamin D levels < 50 nmol/L for all participants and for both ethnic groups, with the participants tested during summer and autumn having the highest serum levels. The British/Irish had higher 25(OH)D levels (above 50 nmol/L on average in both summer and autumn) than did the Greeks/Cypriots. The two ethnic groups differentiated in that the third-highest levels were found during spring for the British/Irish and in autumn for the Greeks/Cypriots. This could be explained by the Greeks/Cypriots having the second-largest groups of participants in winter compared to the British/Irish that had the least participants during the winter. Other than sun exposure during the summer in the UK, this could also possibly be explained by the Greek/Cypriot group traveling back to countries of origin more often during the year, thus producing enough vitamin D to have a gradual decrease during autumn and winter compared to the British/Irish that have a larger drop between autumn and winter.

A plethora of works in the literature support the findings of this current study that vitamin D increases during summer and autumn in both the UK and Greek population and that season is significantly associated with vitamin D serum levels [11,38,45]. A study in Greeks found 39.3% of participants had 25(OH)D between 30–49.9 nmol/L during winter, 25.6% during spring and 25% during summer [45].

### 4.2. Strengths and Limitations

A strength of this work is that it is the first (to the authors’ knowledge) study assessing 25(OH)D concentrations in UK dwelling Greek and Cypriot people. Also, the data were drawn from a highly respected cohort study. One of the limitations is the smaller sample of Greek/Cypriot participants compared to the British/Irish ethnic group, as well as the slight difference in seasons that the blood draws of the ethnic groups were taken in. The ethnic groups may differ in the duration of time spent in sunnier/lower-latitude climates, particularly with respect to the Greek/Cypriot participants potentially returning regularly to Greece/Cyprus. However, the time spent in Greece/Cyprus may not differ from that of the British/Irish group, who may also have been on holiday in similar climates. We were not able to analyse holidays/other time spent abroad as a potential confounder in the analysis, as it was not a question asked by the UK Biobank at baseline. Similarly, we did not have information on clothing styles, so were not able to assess this as a confounder.

Also, the UK Biobank participants are not necessarily representative of the general population. They are likely to be more health conscious/scientifically aware. One published paper found that UK Biobank participants were, on average, more likely to live in more-deprived areas, be female, be healthier and be older than the general population [57]. In terms of sample sizes, the Greek/Cypriot group was smaller than the British/Irish group, which may impact the representativeness of the Greek/Cypriot group, as well as statistical power for subgroup analyses. Larger sample sizes in future studies would help address this issue. In addition, there were more female participants in the British/Irish group and more males in the Greek/Cypriot group. Due to the mean age of 57–58 years, the results of this study cannot be generalised to all age groups.

Missing data was a potential issue, particularly for dietary intakes. For example, 59% of Greeks/Cypriots and 57% of British/Irish did not have data on vitamin D intake. This means that there was likely to be some degree of selection bias, with perhaps healthier, wealthier or more motivated individuals represented more in the dataset than those with poorer health, less wealth, or less motivation. Email reminders for questionnaire completion (with only online completion) may exclude those with less technological ability or access. Not having all participants completing all questionnaires will affect both the statistical power and generalisability of the results, and our results may be considered a best-case scenario, with a significant possibility of even lower vitamin D intakes in the real population of Greek and Cypriot persons in the UK. Results from a UK population may not apply to other populations. Measurement of vitamin D intake was by 24 h dietary recall (FFQ), so not done by the gold standard method of weighted food records. The FFQ only included foods that were consumed in the last 24 h, so foods such as oily fish (that are known to be consumed only on some days during the week or month) may have been excluded but may represent important contributors to vitamin D intake. This could lead to an underestimation of vitamin D intake in this study. Some studies have shown that food diaries are not representative of actual intake due to recall bias as well as under- and overestimation of portion sizes [58]. These limitations would apply to the FFQ used in this study.

Due to the large sample size, the analysis may be overpowered (to detect a difference of 5 nmol/L, *n* = 278 in each group was required for 80% power).

It was not specified which brands of supplement were used and how much vitamin D they contained. Additionally, these participants could be using cod liver oil supplements or prescribed vitamin D medications, which were not assessed here. Additionally, participants were not asked if they had travelled in the past few months. This would have been extremely important for the Greeks/Cypriots who may have travelled back for visits to Greece or Cyprus, and for the British/Irish who may have travelled to sunny countries during annual leave.

Finally, the DiaSorin Liaison XL assay was used to assess 25-hydroxyvitamin D levels. This assay is known to underestimate vitamin D serum levels compared to the gold standard (LC-MS) [32,59,60]. This may affect the generalisability of our results.

## 5. Conclusions

Our findings have important implications for UK dwelling populations from southern Europe. The Greeks/Cypriots had lower 25(OH) levels by 7.4 nmol/L compared to the British/Irish ethic group, although the logistic regression analysis suggested this may be due to other factors related to ethnicity, not ethnicity per se. This still indicates that the Greeks/Cypriots may be a UK ethnic minority group at higher risk of vitamin D deficiency. A large number of participants in both ethnic groups did not meet SACN [38] and EFSA [37] aims for population intake or vitamin D status. To our knowledge, in Greece and Cyprus, there are no recommendations for 25(OH)D serum levels or vitamin D intakes, and no mandatory food supplementation for vitamin D [17,24]. This may result in a lack of awareness from this ethnic group regarding the risk of vitamin D deficiency.

Previous studies conducted in Greece and Cyprus have shown a high prevalence of vitamin D deficiency, low vitamin D intake and low use of supplements. The results of our current analysis show similar findings for this ethnic group living in the UK. One concern is that they are at even higher risk of vitamin D deficiency in the UK than when living in their country of origin, and when compared to the British/Irish ethnic group, as the UK is a country where there is even less vitamin D production in the skin during the winter months.

Public health policies and campaigns should further support improvement in vitamin D intake across the UK population, with a focus on ethnic minorities, including those originating from southern European countries, especially those who have pigmented skin and who do not get enough sun exposure.

## Figures and Tables

**Table 1:** Summarizing data for the continuous non-normally distributed variables.

Variables	All Participants (*n* 4483)				British/Irish (*n* 4158)				Greek/Cypriot (*n* 325)				* *p*
	Median	IQR	*n*	Missing	Median	IQR	*n*	Missing	Median	IQR	*n*	Missing	
25(OH)D status baseline nmol/L	47.10	30.9	3996	487	47.60	31.3	3707	451	40.30	21.5	289	36	<0.001
Vitamin D intake median over visits μg/day	1.76	2.02	1911	2572	1.77	2.02	1779	2379	1.41	1.82	132	193	0.045
Age at baseline assessment (years)	58.00	12	4483	0	58.00	12	4158	0	57.00	12	325	0	0.004
Townsend Deprivation Index	−2.24	4	4480	3	−2.31	4	4155	3	−0.66	4	325	0	<0.001
Age completed Full time education	16.00	2	2964	1519	16.00	2	2820	1338	17.00	4	144	181	<0.001

* Data included for all participants and split by ethnicity together with the *p* value generated from the Mann-Whitney Test.

**Table 2:** Summarizing the results of the chi-square test assessing categorical variables for all participants and split by ethnicity.

		British/Irish		Greek/Cypriot		Chi-Square
		%	*n*	%	*n*	*p*
Gender						
	Female	55.7	2315	47.1	153	0.003
	Male	44.3	1843	52.9	172	
Household income						
	Less than 18,000	23.7	848	21.9	56	<0.001
	18,000–30,999	25.8	924	19.1	49	
	31,000–51,999	25.9	925	21.5	55	
	52,000–100,000	19.4	695	27.3	70	
	>100,000	5.2	186	10.2	26	
Oily Fish Intake						
	never	10.8	448	4.4	14	0.02
	Less than once a week	32.2	1332	37.1	119	
	Once per week	39.8	1644	40.5	130	
	2–4 times per week	16.3	672	16.5	53	
	5–6 times per week	0.6	24	1.6	5	
	Once or more daily	0.3	11	0.0	0	
Alcohol Consumption						
	Almost daily or daily	20.7	858	11.1	36	<0.001
	3–4 times per week	24.0	995	16.4	53	
	1–2 times per week	27.1	1125	22.6	73	
	1–3 times per month	11.3	468	11.8	38	
	Special occasions only	10.4	430	27.6	89	
	never	6.7	278	10.5	34	
Skin Colour						
	Very fair	8.3	336	1.9	9	<0.001
	Fair	72.1	2950	28.0	89	
	Light Olive	18.2	746	59.4	189	
	Dark Olive	1.0	41	5.7	18	
	Brown	0.3	13	5.0	16	
Smoking Status						
	Never	54.6	2261	45.2	145	<0.001
	Previous	34.7	1437	37.1	119	
	Current	10.7	443	17.8	57	
User of Single Vit D supplement or multivitamin/mineral						
	User	23.6	970	28.4	91	0.051
	Non user	76.4	3141	71.6	229	
Region of assessment						
	Northern England	47.3	1968	16.6	54	<0.001
	Southern England	16.8	698	17.8	58	
	Wales	1.3	53	3.1	10	
	Scotland	8.1	335	2.2	7	
	English Midlands	16.0	667	12.9	42	
	London	10.5	437	47.4	154	
Summer sun exposure per day						
	Less than 30 min	4.1	159	8.1	24	0.001
	More than 30 min	95.9	3730	91.9	273	
Season						
	Spring	28.4	1180	36.0	117	0.019
	Summer	26.1	1084	20.6	67	
	Winter	24.2	1007	23.7	77	
	Autumn	21.3	887	19.7	64	

**Table 3:** Summarizing the number and percentage of participants found to have serum levels above and below 25 nmol/L and 50 nmol/L.

	All Participants		British/Irish		Greeks/Cypriots		*p*
	*N*	%	*n*	%	*n*	%	
≥25 nmol/L	3521	88.1	3298	89.0	223	77.2	<0.001
<25 nmol/L	475	11.9	409	11.0	66	22.8	
≥50 nmol/L	1848	46.2	1739	46.9	109	37.7	<0.003
<50 nmol/L	2148	53.8	1968	53.1	180	62.3	

## Data Availability

Restrictions apply to the availability of these data. Data were obtained from UK Biobank and are available [see https://www.ukbiobank.ac.uk/use-our-data/fees/, accessed on 7 May 2025] with the permission of UK Biobank.

## Supplementary Materials

The following supporting information can be downloaded at: https://www.mdpi.com/article/10.3390/nu17203267/s1, Table S1. Summarizing the results of the logistic regression model caried out for all participants investigating the odds of having 25(OH)D serum levels > 50 nmol/L; Table S2. Summarizing the results of the logistic regression model caried out for the British/Irish ethnic group investigating the odds of having 25(OH)D serum levels > 50 nmol/L; Table S3. Summarizing the results of the logistic regression model caried out for the Greek/Cypriot ethnic group investigating the odds of having 25(OH)D serum levels > 50 nmol/L.
