# Supplementary material for: 25-Hydroxyvitamin D Status and Its Predictors in Greek and Cypriot Subsets of the UK Biobank Cohort
Source: Nutrients. 2025 Oct 17;17(20):3267. doi: 10.3390/nu17203267 (PMC12567200; doi:10.3390/nu17203267)
Supplement: Supplementary file 1 [file nutrients-17-03267-s001.zip › nutrients-3719200-supplementary.pdf]

25-hydroxyvitamin D status and its predictors in Greek and Cypriot subsets of the UK biobank Cohort

Francesca E. Kontea, Susan A. Lanham-New and Andrea L. Darling

Supplementary Table S1. Summarizing the results of the logistic regression model caried out for all participants investigating the odds of having 25(OH)D serum levels >50nmol/L.

		n	B	SE	Wald	df	p	OR	Lower 95% CI	Upper 95% CI
**Sex**										
	Female	**1676**						**1.0**		
	Male	**1555**	**0.008**	**0.08**	**0.009**	**1**	**0.924**	**1.008**	**0.862**	**1.178**
**Household income before tax**										
	Less than 18.000	**750**			**5.07**	**4**	**0.28**	**1.0**		
	£18,000 to 30,999	**786**	**-0.178**	**0.113**	**2.486**	**1**	**0.115**	**0.837**	**0.67**	**1.044**
	£31,000 to 51,999	**840**	**-0.036**	**0.113**	**0.102**	**1**	**0.75**	**0.965**	**0.773**	**1.204**
	£52,000 to 100,000	**670**	**-0.042**	**0.121**	**0.118**	**1**	**0.731**	**0.959**	**0.756**	**1.217**
	Greater than 100,000	**185**	**-0.309**	**0.186**	**2.773**	**1**	**0.096**	**0.734**	**0.51**	**1.056**
**Oily Fish Intake**										
	Never	**328**			**4.618**	**5**	**0.464**	**1.0**		
	Less than once per week	**1065**	**-0.059**	**0.14**	**0.179**	**1**	**0.672**	**0.942**	**0.716**	**1.24**
	Once per week	**1304**	**-0.199**	**0.137**	**2.1**	**1**	**0.147**	**0.819**	**0.626**	**1.073**
	2-4 times per week	**502**	**-0.234**	**0.157**	**2.226**	**1**	**0.136**	**0.791**	**0.581**	**1.076**
	5-6 times per week	**25**	**-0.045**	**0.461**	**0.01**	**1**	**0.922**	**0.956**	**0.387**	**2.361**
	Once or more daily	**7**	**-21.275**	**14579.88**	**0**	**1**	**0.999**	**0**	**0**	**.**
**Alcohol Consumption**										
	Daily or almost daily	**658**			**14.872**	**5**	**0.011**	**1.0**		
	3-4 times per week	**803**	**-0.185**	**0.115**	**2.569**	**1**	**0.109**	**0.831**	**0.663**	**1.042**
	1-2 times per week	**858**	**-0.011**	**0.116**	**0.01**	**1**	**0.921**	**0.989**	**0.788**	**1.24**
	1-3 times per month	**363**	**0.195**	**0.146**	**1.763**	**1**	**0.184**	**1.215**	**0.912**	**1.619**

	Special occasions only	**341**	**0.297**	**0.155**	**3.684**	**1**	**0.055**	**1.345**	**0.994**	**1.821**
	Never	**208**	**0.212**	**0.179**	**1.395**	**1**	**0.237**	**1.236**	**0.87**	**1.756**
**Smoking status**										
	Never	**1724**			**2.907**	**2**	**0.234**	**1.0**		
	Previous	**1153**	**-0.001**	**0.085**	**0**	**1**	**0.991**	**0.999**	**0.846**	**1.18**
	Current	**354**	**0.215**	**0.132**	**2.676**	**1**	**0.102**	**1.24**	**0.958**	**1.605**
**User of single Vitamin D supplement or a multivitamin/mineral**										
	User	**774**						**1.0**		
	Non-user	**2457**	**0.877**	**0.092**	**90.15**	**1**	**<.001**	**2.404**	**2.006**	**2.882**
**Region of Assessment center**										
	North England	**1433**			**7.478**	**5**	**0.187**	**1.0**		
	South England	**586**	**-0.004**	**0.108**	**0.001**	**1**	**0.969**	**0.996**	**0.805**	**1.232**
	Wales	**47**	**0.034**	**0.331**	**0.01**	**1**	**0.919**	**1.034**	**0.541**	**1.979**
	Scotland	**259**	**0.404**	**0.156**	**6.728**	**1**	**0.009**	**1.498**	**1.104**	**2.032**
	English Midlands	**484**	**-0.029**	**0.116**	**0.063**	**1**	**0.802**	**0.971**	**0.774**	**1.218**
	London	**422**	**0.007**	**0.128**	**0.003**	**1**	**0.959**	**1.007**	**0.784**	**1.293**
**Summer sun per day**										
	less than 30 minutes	**141**						**1.0**		
	Over 30 minutes	**3090**	**-0.826**	**0.201**	**16.846**	**1**	**<.001**	**0.438**	**0.295**	**0.649**
**Season**										
	Spring	**936**			**330.472**	**3**	**<.001**	**1.0**		
	Summer	**833**	**-1.565**	**0.107**	**213.521**	**1**	**<.001**	**0.209**	**0.169**	**0.258**
	Autumn	**802**	**-1.136**	**0.105**	**116.827**	**1**	**<.001**	**0.321**	**0.261**	**0.395**
	Winter	**660**	**0.165**	**0.116**	**2.034**	**1**	**0.154**	**1.179**	**0.94**	**1.479**
**Ethnicity**										
	British or Irish	**3015**						**1.0**		
	Greece or Cyprus	**216**	**0.161**	**0.167**	**0.937**	**1**	**0.333**	**1.175**	**0.848**	**1.629**
	Constant		**1.088**	**0.279**	**15.191**	**1**	**<.001**	**2.969**		

Supplementary Table S2. Summarizing the results of the logistic regression model caried out for the British/Irish ethnic group investigating the odds of having 25(OH)D serum levels >50nmol/L.

		n	B	SE	Wald	df	p	OR	Lower 95% CI	Upper 95% CI
Sex										
	Female	1575						1.0		
	Male	1440	**-0.016**	**0.082**	**0.036**	**1**	**0.849**	**0.984**	**0.838**	**1.157**
Household income before tax										
	Less than 18.000	700			**6.618**	**4**	**0.158**	1.0		
	£18,000 to 30,999	750	**-0.183**	**0.116**	**2.459**	**1**	**0.117**	**0.833**	**0.663**	**1.047**
	£31,000 to 51,999	793	**-0.009**	**0.117**	**0.006**	**1**	**0.938**	**0.991**	**0.788**	**1.246**
	£52,000 to 100,000	611	**0.006**	**0.126**	**0.002**	**1**	**0.964**	**1.006**	**0.785**	**1.288**
	Greater than 100,000	161	**-0.35**	**0.197**	**3.143**	**1**	**0.076**	**0.705**	**0.478**	**1.038**
Oily Fish Intake										
	Never	317			**6.985**	**5**	**0.222**	1.0		
	Less than once per week	980	**-0.08**	**0.143**	**0.31**	**1**	**0.578**	**0.923**	**0.697**	**1.223**
	Once per week	1222	**-0.232**	**0.14**	**2.721**	**1**	**0.099**	**0.793**	**0.603**	**1.045**
	2-4 times per week	468	**-0.288**	**0.161**	**3.214**	**1**	**0.073**	**0.749**	**0.547**	**1.027**
	5-6 times per week	21	**0.379**	**0.511**	**0.551**	**1**	**0.458**	**1.461**	**0.537**	**3.978**
	Once or more daily	7	**-21.334**	**14529.22**	**0**	**1**	**0.999**	**0**	**0**	**.**
Alcohol Consumption										
	Daily or almost daily	631			**15.735**	**5**	**0.008**	1.0		
	3-4 times per week	769	**-0.208**	**0.118**	**3.117**	**1**	**0.077**	**0.812**	**0.645**	**1.023**
	1-2 times per week	807	**0.001**	**0.119**	**0**	**1**	**0.993**	**1.001**	**0.794**	**1.263**
	1-3 times per month	338	**0.169**	**0.151**	**1.251**	**1**	**0.263**	**1.184**	**0.881**	**1.591**
	Special occasions only	282	**0.31**	**0.164**	**3.599**	**1**	**0.058**	**1.364**	**0.99**	**1.88**
	Never	188	**0.261**	**0.186**	**1.96**	**1**	**0.162**	**1.298**	**0.901**	**1.871**
Smoking status										
	Never	1632			**3.48**	**2**	**0.175**	1.0		
	Previous	1066	**-0.048**	**0.088**	**0.296**	**1**	**0.587**	**0.954**	**0.803**	**1.132**
	Current	317	**0.218**	**0.138**	**2.483**	**1**	**0.115**	**1.243**	**0.948**	**1.63**
User of single Vitamin D supplement or a multivitamin/mineral										

	User	709						**1.0**		
	Non-user	2306	**0.867**	**0.096**	**81.052**	**1**	**<.001**	**2.379**	**1.97**	**2.874**
Region of Assessment center										
	North England	1395			**5.773**	**5**	**0.329**	1.0		
	South England	542	**0.017**	**0.111**	**0.025**	**1**	**0.875**	**1.018**	**0.818**	**1.266**
	Wales	41	**0.028**	**0.352**	**0.006**	**1**	**0.937**	**1.028**	**0.516**	**2.049**
	Scotland	254	**0.362**	**0.157**	**5.319**	**1**	**0.021**	**1.436**	**1.056**	**1.954**
	English Midlands	462	**-0.025**	**0.118**	**0.044**	**1**	**0.834**	**0.976**	**0.774**	**1.23**
	London	321	**0.027**	**0.137**	**0.04**	**1**	**0.842**	**1.028**	**0.785**	**1.345**
Sumer sun per day										
	less than 30 minutes	123						**1.0**		
	Over 30 minutes	2892	**-0.745**	**0.212**	**12.33**	**1**	**<.001**	**0.475**	**0.313**	**0.719**
Season										
	Spring	852			**310.613**	**3**	**<.001**	1.0		
	Summer	791	**-1.551**	**0.111**	**196.268**	**1**	**<.001**	**0.212**	**0.171**	**0.263**
	Autumn	749	**-1.094**	**0.109**	**100.975**	**1**	**<.001**	**0.335**	**0.27**	**0.414**
	Winter	623	**0.227**	**0.12**	**3.592**	**1**	**0.058**	**1.255**	**0.992**	**1.588**
Constant			**1.029**	**0.291**	**12.483**	**1**	**<.001**	**2.799**		

Supplementary Table S3. Summarizing the results of the logistic regression model caried out for the Greek/Cypriot ethnic group investigating the odds of having 25(OH)D serum levels >50nmol/L

		**n**	**B**	**SE**	**Wald**	**df**	**p**	**OR**	**Lower 95% CI**	**Upper 95% CI**
Sex										
	Female	101						1.0		
	Male	115	**0.23**	**0.369**	**0.389**	**1**	**0.533**	**1.259**	**0.611**	**2.592**
Household income before tax										
	Less than 18.000	50			**2.798**	**4**	**0.592**	1.0		
	£18,000 to 30,999	36	**0.204**	**0.576**	**0.125**	**1**	**0.724**	**1.226**	**0.396**	**3.793**
	£31,000 to 51,999	47	**-0.318**	**0.545**	**0.342**	**1**	**0.559**	**0.727**	**0.25**	**2.115**
	£52,000 to 100,000	59	**-0.604**	**0.523**	**1.335**	**1**	**0.248**	**0.546**	**0.196**	**1.523**
	Greater than 100,000	24	**-0.535**	**0.683**	**0.612**	**1**	**0.434**	**0.586**	**0.154**	**2.236**
Oily Fish Intake										
	Never	11			**6.458**	**4**	**0.167**	1.0		
	Less than once per week	85	**1.236**	**0.832**	**2.208**	**1**	**0.137**	**3.44**	**0.674**	**17.554**

	Once per week	82	1.294	0.839	2.38	1	0.123	3.647	0.705	18.871
	2-4 times per week	34	1.689	0.905	3.486	1	0.062	5.416	0.919	31.901
	5-6 times per week	4	-1.511	1.541	0.962	1	0.327	0.221	0.011	4.519
	Once or more Daily		Not estimable							
Alcohol Consumption										
	Daily or almost daily	27			3.602	5	0.608	1.0		
	3-4 times per week	34	0.46	0.624	0.543	1	0.461	1.584	0.466	5.385
	1-2 times per week	51	-0.212	0.6	0.124	1	0.724	0.809	0.25	2.622
	1-3 times per month	25	0.789	0.692	1.301	1	0.254	2.201	0.567	8.541
	Special occasions only	59	0.429	0.602	0.506	1	0.477	1.535	0.471	4.998
	Never	20	0.242	0.787	0.095	1	0.758	1.274	0.272	5.964
Smoking status										
	Never	92			4.145	2	0.126	1.0		
	Previous	87	0.863	0.424	4.143	1	0.042	2.371	1.033	5.443
	Current	37	0.387	0.511	0.574	1	0.449	1.473	0.541	4.008
User of single Vitamin D supplement or a multivitamin/mineral										
	User	65						1.0		
	Non-user	151	1.118	0.373	8.979	1	0.003	3.057	1.472	6.351
Region of Assessment center										
	North England	38			0.231	5	0.999	1.0		
	South England	44	-0.045	0.6	0.006	1	0.941	0.956	0.295	3.101
	Wales	6	0.449	1.103	0.166	1	0.684	1.567	0.18	13.615
	Scotland	5	20.84	16280.09	0	1	0.999	1.12E+09	0	.
	English Midlands	22	-0.09	0.704	0.016	1	0.899	0.914	0.23	3.636
	London	101	-0.044	0.518	0.007	1	0.932	0.957	0.347	2.643
Sumer sun per day										
	less than 30 minutes	18						1.0		
	Over 30 minutes	198	-2.08	0.824	6.371	1	0.012	0.125	0.025	0.628
Season										
	Spring	84			22.959	3	<.001	1.0		
	Summer	42	-2.067	0.5	17.086	1	<.001	0.127	0.047	0.337
	Autumn	53	-1.783	0.465	14.731	1	<.001	0.168	0.068	0.418
	Winter	37	-0.503	0.497	1.026	1	0.311	0.604	0.228	1.601

Constant	**0.848**	**1.289**	**0.433**	**1**	**0.511**	**2.335**
